# Younger Adolescents’ Perceptions of Physical Activity, Exergaming, and Virtual Reality: Qualitative Intervention Development Study

**DOI:** 10.2196/11960

**Published:** 2019-06-17

**Authors:** Nuša Faric, Eleanor Yorke, Laura Varnes, Katie Newby, Henry WW Potts, Lee Smith, Adrian Hon, Andrew Steptoe, Abigail Fisher

**Affiliations:** 1 Department of Behavioural Science & Health University College London London United Kingdom; 2 Faculty Research Centre for Advances in Behavioural Science Coventry University Coventry United Kingdom; 3 Institute of Health Informatics University College London London United Kingdom; 4 Cambridge Centre for Sport and Exercise Sciences Anglia Ruskin University Cambridge United Kingdom; 5 Six to Start London United Kingdom

**Keywords:** exercise, obesity, video games, adolescent, adolescence, sports, health, leisure activities, virtual reality

## Abstract

**Background:**

Novel strategies to promote physical activity (PA) in adolescence are required. The vEngage study aims to test whether a virtual reality (VR) exergaming intervention can engage younger adolescents (aged 13 to 15 years) with PA.

**Objective:**

This study aimed to gather adolescents’ views of using VR to encourage PA and identify the key features they would like to see in a VR exergaming intervention via interviews.

**Methods:**

Participants were recruited through 2 schools in London, United Kingdom. Semistructured interviews were conducted with adolescents about their views on PA and what might work to increase PA, technology, knowledge and experience of VR, and desired features in a VR exergaming intervention. Data were analyzed using Framework Analysis.

**Results:**

A total of 31 participants aged between 13 and 15 years (58% female, 62% from nonwhite ethnicities) participated in this interview study. The vast majority had no awareness of government PA recommendations but felt they should be more thoroughly informed. All participants were positive about the use of VR in PA promotion. Rewards, increasing challenges, and a social or multiplayer aspect were identified by participants as crucial aspects to include in a VR exercise game. Barriers were related to cost of high-end systems. Being able to exercise at home was very appealing. VR exergaming was viewed as a way to overcome multiple perceived social and cultural barriers to PA, particularly for girls.

**Conclusions:**

Key elements that should be incorporated into a VR game for health intervention were identified and described. These also included the use of rewards, novelty and enjoyment in immersive game play, multiplayer options, and real-world elements, as well as continual updates and new challenge levels. The use of VR to promote PA in adolescents is promising, but some barriers were raised.

## Introduction

### Limited Effectiveness of Physical Activity Interventions in Adolescents

The health benefits of performing sufficient physical activity (PA) are well established and include reduced risk of noncommunicable diseases, reduced risk of premature mortality, and better mental health [1-3]. There is a dose-response relationship between PA and health with a 20% to 30% reduction in chronic illness and premature death for those that meet PA guidelines [1-3]. Adolescence (13 to 17 years) is a key time to intervene, as long-term PA likely confers maximum protective benefit [3]. Those who have high levels of PA in adolescence are more likely to be active in adulthood and lead healthier lifestyles [4,5]. There are psychological and social benefits of increased PA participation in adolescence [6]. However, less than 15% of boys and 10% of girls are achieving the UK government recommendation for adolescents of at least 60 min of moderate-to-vigorous physical activity (MVPA) per day [7]. Levels of adolescent PA are similarly low in other developed countries [8]. Without intervention, activity levels decline by around 7% per year throughout adolescence, particularly in girls [9]. Strategies to increase PA in this group are urgently required.

It remains unclear what works best to change adolescent PA behavior [10,11]. A recent review of digital PA interventions for adolescents recommended that education, goal setting and feedback, self-monitoring, and parental support should be incorporated [12].

Despite recommendation that conducting formative work with target users is important to intervention development [13,14], very few PA interventions have involved adolescents in development. Before developing a school-based intervention, Corder et al conducted focus groups with participants aged between 16 and 18 years and identified choice, novelty, mentorship, rewards, competition, and flexibility as key aspects that young people would like to be included [15]. Although co-design or participatory design (PD) of digital health interventions is not necessarily recommended (as no higher effectiveness was found for games developed with PD), user input is beneficial [16]. This is particularly applicable when considering digital PA interventions [16].

### Digital Physical Activity Interventions in Adolescents

Digital interventions are likely to be particularly appealing for adolescents; more than 90% play video games for at least an hour per day [17]. A variety of exergames are available and many have been commercial successes: Wii Fit sold over 22 million copies worldwide in its first 4 years [18]. More recently, an augmented reality exergame run on smartphones, Pokémon Go, has seen over 800 million downloads [19]. A study conducted in Hong Kong (participants N=210; aged 16 to 64 years) found that the use of Pokémon Go was associated with a short-term increase in the players’ daily walking and running distances, particularly in those who were less physically active [20].

Levels of PA and physiological response when exergaming are comparable with field-based PA, and significantly increased when compared with standard gaming [21]. Exergames also enhanced enjoyment, self-efficacy, and motivation for PA [21]. Exergaming interventions can lead to weight loss in overweight adolescents [22,23]. However, past research has generally involved small studies and earlier generation exergames.

Virtual reality (VR) has the potential to enhance the exergaming experience through immersion and presence contributing to the feeling of absorption, flow, and fun [23,24]. New generation VR technologies deliver increasingly realistic experiences at decreasing cost. Some small laboratory-based studies in adults have found that immersive VR exergames resulted in the same or higher intensity of exercise as standard exercise conditions, but with higher ratings of enjoyment and interest [25,26]. One study also found that perceived exertion was lower and self-efficacy was higher during VR cycling compared with standard stationary cycling [27]. An exploratory pre-post study including 9 children and adolescents suggested that an immersive VR game enhanced motivation to be active [28]. Immersion is likely to distract participants from exertion and possibly negative thinking during PA.

We hypothesized that a VR exergaming intervention could increase PA in adolescents. The aim of this study was to interview adolescents about their recommendations for a PA intervention, use of technology, gaming, and interest in VR for PA.

## Methods

### Participants

Participants were recruited from 2 secondary schools in London, United Kingdom between January and July 2017. To be eligible, participants had to be aged between 13 and 17 years. Given the need to recruit a high number of girls, one was a girl’s school whereas the other was mixed. Both schools were in areas of high ethnic and socioeconomic diversity. Each school sent information packs home with students from 2 classes. In total, approximately 65 packs were sent to the interested students. Interested students each returned and completed the parental consent and child assent form. We aimed to interview approximately 30 young people based on guidance provided by Fugard and Potts [29] and we interviewed all students who returned the completed consent forms.

### Ethics

Ethical approval was provided by the University College London Ethics committee (Project ID 10213/001) with all participants and their parents/caregivers providing informed written consent.

### Qualitative Interviews

We used a semistructured interview. We asked the following: “What is the amount of time you spend being active as well as inactive per week in hours?”; “What is your average sitting time per day?”; “Do you know about the recommended guidelines for PA in your age group?”; What might encourage people of your age to become more physically active?”; “What is your current technology use- what do you use and how long per day or week?”; “What interest you about a particular game?”; “Do you know much about VR?”; “What are your experiences and/or opinion on using VR?”; “Do you use any other health-related technology?”; and “How would you help increase PA through use of technology and VR?”. Finally, participants were asked about key features desired in a VR exergame. Interviews were conducted face-to-face in schools between June and July 2017 by 2 researchers (EY and LV) and transcribed verbatim.

### Analysis

Framework Analysis was used, which is a flexible and systematic approach for analysis of semistructured interviews [30]. A total of 2 researchers (EY and LV) independently analyzed 3 transcripts, each developing an initial set of codes. The participant’s responses within the interviews were assigned codes and coded into a framework matrix, along with relevant quotes to make it visually straightforward and easy to track. Then 2 additional researchers (NF and AF) independently interpreted the data to identify common themes among responses. Theme prevalence was not determined quantitatively but instead grouped together into main themes described below. All researchers then met and discussed their interpretations and codes, compared them, and made minor adjustments to create the final framework. The final interpretations are shared below, with illustrative quotes (followed by participant’s gender and age).

## Results

### Overview

A total of 31 participants aged between 13 and 15 years were interviewed. Of them, 58% (18/31) were girls. Participants identified their ethnicity as white British (n=7), white other (n=5), multiple ethnicities (n=5), Asian (n=5), Indian (n=2), Bangladeshi (n=2), Pakistani (n=1), Caribbean (n=1), African (n=1), and black (n=2). Interviews lasted for 40 to 70 min (mean 55 min). The themes were apparent and straightforward with no disagreements between the researchers. The main themes and their subthemes are described in Table 1.

**Table 1:** Identified main themes and subthemes.

Main theme	Subthemes
PA^a^ and sedentary time	Adolescents were not aware of the PA guidelines for people of their age or of all health benefits associated with PA or consequences of not performing sufficient PA
General technology use	Smartphones were the technology most used by adolescents for recreation; Gaming was popular and exergames were a positive past experience, but some games were no longer played (eg, Just Dance or Pokémon Go)
Exergaming	Exergames were seen as a fun, motivating, and encouraging movement covertly; There was a strong appeal of exercising at home and overcoming cultural or social barriers, particularly for girls; Exergames were not seen as a replacement if already involved in sport
Experience of VR^b^	Positivity toward VR but limited experience; Whole body movement, presence, and novelty appealing in VR; Barriers with VR included bulky headsets, addiction, and price; Perceived parental concern about using VR for PA; Simple public health messages about screen time preferred

^a^PA: physical activity.

^b^VR: virtual reality.

### Physical Activity and Sedentary Time

#### Adolescents Were Not Aware of the Recommended Guidelines for Physical Activity and of All Health Benefits and Risks Associated With Physical Activity

Awareness of PA recommendations for adolescents was very low with 94% (29/31) participants guessing the recommended guidelines. The intensity or resistance exercises were not mentioned. Participants felt strongly that people of their age *should* be informed of PA recommendations and the links between PA and health. Girls in particular reported this knowledge as a key motivation for being active and felt this would increase activity in their peers:

Give [young people] some of the risks that could happen in the future if you do not keep fit and not healthy.

F, 14

When probed about how they might like this information delivered, the consensus was visually:

Maybe videos on how to do it because I don't think people [my age] really take in facts.

F, 14

But tangible rewards were also important motivators for PA:

So there’s something to work for instead of just saying do it and you’ll be more active. You have to give them something at the end. Anything people my age will find fun. Probably mostly money.

M, 15

### General Technology Use

#### Mainly Smartphones, Mainly for Recreation

All participants used technology up to 6 hours per day (eg, smartphone and computer), mainly for recreational uses (eg, gaming and social media) but also practical (eg, homework). Boys used technology mainly for gaming, whereas girls used technology for watching videos, listening to music, and socializing (reported logging into social media between 30 and 55 times per day). There were positive and negative perceptions of technology related to PA:

I saw this report, kids nowadays are more overweight compared to before, and that's probably because we're always just sitting down on our phones, tablets and stuff.

F, 14

[use technology to encourage people to go outside] because obviously with technology people don’t go outside as much as they used to. So, I don’t know, you just need to get people out. Because the phone is so interesting.

F, 15

#### Gaming was Popular and Exergames Were a Positive Past Experience

The majority of participants engaged in some type of gaming. Games played were diverse, but common features were continual challenges/levels of difficulty, rewards, competition, social aspects, and story modes. A total of 5 participants specifically mentioned games that were simple, slow to build, and ongoing as leading to playing for a number of years. Exergames were often mentioned; all participants had played them, and they were generally described positively, but always in the past tense such as Just Dance (Nintendo Wii):

I really liked that [Just Dance] but I don’t know, it just died out for some reason, I don’t know why. We had the dancing thing, to step on it, all that kind of stuff, yes. Yes, that was sick.

F, 15

#### Pokémon, Gone

Many participants described Pokémon Go (released in July 2016) as something they had tried but no longer used, with some stating technical reasons as off-putting:

It was really fun in the beginning, but then the servers were overwhelmed with too many people playing.

M, 14

[I played it] only once then found out it used up most of my data.

F, 13

Many mentioned safety concerns preventing use (Web-based security, road safety, and getting lost), often reflecting on negative stories they had heard in the media. Others felt it had increased their PA, but still referred to it is as a previous experience:

Well, at the time where it was big it was really cool because it actually made me go outside and look around and stuff. It did made me walk a lot more.

F, 14

#### Exergames: Fun, Motivating, and Encouraging Movement Covertly

Exergames were viewed as appealing because they were fun, motivating, and good ways to encourage incidental PA:

I think it’s good because some people may think that, “I don't want to do this sport,” but actually, they are, without realising.

F, 15

It’s way better because you’re working out but you’re having fun and you don't realise you’re working out.

F, 14

Social benefits and competition when exergaming were also described as appealing:

It’s nice because other people can actually watch it at the same time, it’s not just one person involved in it. So, it makes it fun and motivates the person to even try harder, so it creates competition as well.

F, 15

#### Strong Appeal of Exercising at Home and Overcoming Cultural or Social Barriers

Participants particularly liked the idea that exergames allowed activity in the home:

Getting fit isn’t always enjoyable and it could also make you confident to do it[...] You can do it in the privacy of your own home.

F, 14

Virtual reality...in your own home because people like going running but sometimes people can’t...my parents don’t always let me out, so doing it in my own home...Yes. And also for Muslims, you have to cover your body, so it’s hard to go running while covering your body, whereas at home it’s very easy.

F, 14

#### Exergames Not a Replacement if Already Involved in Sport

However, those who were already involved in sport tended to think exergames should not be a replacement:

I think they're useful, but I think it's better for people to actually do sport.

F, 14

### Experience of Virtual Reality

#### Positivity Toward Virtual Reality but Limited Experience

Nearly all participants were extremely positive about the idea of using or trying VR. More than half had tried some kind of VR (usually smartphone-based headsets). There were no apparent gender differences in wanting to try, having tried, or liking VR. A total of 6 participants described having tried a one-off fully immersive experience in an external venue, and 1 had high-end equipment at home. Very few had any kind of VR equipment at home, and if they did, it was usually referred to as being owned by a parent or older sibling. Participants wanted to own it but felt the price was prohibitive:

I've used it once when I went to a big shopping centre. I thought it was quite cool but then I looked at the price: thousands.

M, 15

#### Whole Body Movement, Presence, and Novelty Appealing in Virtual Reality

Those who had tried VR were positive about it, describing it frequently as *cool*, *exciting*, *fun,* and highlighting the whole body movement, presence, and novelty as appealing. Only 1 participant thought it was *pointless* (F, 14).

You’re actually in the game, you can feel you're moving with it, it’s not just your fingers and your eyes, it’s your whole body is involved so it’s more involved.

F, 15

I think it’s absolutely cool. For [named a standard games console], you sit in front of a TV, but now when it actually feels like you're there, it makes it way more interesting and fun.

F, 15

Other benefits were raised such as widening understanding/experience or creating safe spaces to try new activities:

It is like being in real life, but safer, if you know what I mean. Like if you do something serious, it is not real. So, in a way, it is safer to learn things.

F, 14

#### Barriers With Virtual Reality Included Bulky Headsets, Addiction, and Price

There were technical barriers including the size and weight of the headset. Barriers were usually countered with belief that these issues would be addressed as the technology advanced:

I think it’s a great concept, but I think it has a way to go. It’s not really developed as much yet...Because right now you have to wear like a massive headset that’s really heavy and you have to move around and it’s not as receptive of the little controls.

M, 15, owned VR headset

As with other types of gaming, a prominent concern was fear of addiction:

Just as they're addicted to PlayStation and all of that, they would be addicted to this game.

F, 14

Some participants described physical symptoms of use, such as nausea, dizziness, headaches, and fear of bumping into others. However, nearly all believed that VR was going to be extremely popular in the future:

I think virtual reality is the future of technology and it will be more involved in everyday life.

F, 14

#### Perceived Parental Concern About Using Virtual Reality for Physical Activity

Although participants felt positively about a VR exergaming intervention, there was a perception that parents might not be supportive:

Some parents would be really opposing to it. Just because I know some of my friends, their parents don't like them being on electronics at all.

F, 14

Parents wouldn’t buy that for their children because obviously price.

M, 13

#### Simple Messages About Screen Time Preferred

When asked about how to counteract potentially conflicting public health messages around reducing screen time versus introducing an exergaming intervention, the consensus among participants was that providing information would be sufficient. Many said things like *just tell us* (that screen time needs to be limited; M, 15) or suggested that it was sitting rather than screen time that was the issue:

I would say if it's in front of a screen and it does encourage physical activity, it doesn't really matter that it's in front of a screen, as long as you're engaging.

M, 14

In addition, this approach could appeal to gamers, who were perceived to be sedentary:

They’re [gamers] probably more interested in video games, so the virtual reality might encourage them more [to be physically active].

F, 14

This view was supported by participants who identified as gamers:

Because it’s a game. Immediately I hear game, personally, I’m into it already and think it will be more exciting.

M, 13

Additional features that participants reported they would like to see in a VR exergame are shown in Table 2 with supporting quotes.

**Table 2:** Desired features of virtual reality physical activity intervention.

Features to incorporate	Quote(s)
Include smartphone-based elements	“An app [for a PA^a^ intervention], because more people have a phone than virtual reality” (F, 14)
Use a popular accessible activity such as dancing/whole body movement	“I would probably start with just people getting into dancing, because everyone quite likes that and enjoys that” (F, 14); “Something with dancing would be fun, because obviously you have to move your whole body instead of just your arms or your legs” (F, 13)
Regular updates to prevent boredom	“Update it every month so there’s something new every month so it doesn’t get boring” (M, 10)
Break tasks to prevent addiction	“It could give you tasks on there. It could be like, ‘Go outside and find the tallest tree,’ or something. It could encourage you to go outside” (F, 13)
Rewards and prizes	“I would design something like... in the game, if you played the [sport] in real life you’d get a massive prize” (F, 13)
Competition	“Something competitive. People are really competitive in school, so something they can really get into and play as a team” (M, 15)
Multiplayer option	“I would probably try and get all my friends involved first, because you don’t really want to do it alone” (F, 14); “You need it to be with more people. You don’t want to be alone” (F, 13)
Real world elements	“I think it’s a good starting point, definitely for people who can’t go for a run or something. [...] But I would still say going outside is always better” (F, 14)
VR^b^ gaming clubs or meet-ups	“There’d be a club where you bring your VR, and it would be at an affordable price, where you can bring it and do it with people” (F, 13)

^a^PA: physical activity.

^b^VR: virtual reality.

## Discussion

### Principal Findings

Our findings suggest strong support for the potential of VR to promote PA in adolescents. However, there were a number of factors relevant to researchers developing any digital PA intervention discussed below.

Participants raised the importance of parental support, in line with previous research on digital health interventions for adolescents and studies exploring determinants of adolescent PA [12]. We are separately interviewing parents and teachers about their perceptions of VR exergaming and PA.

Awareness of any government guidelines around PA was low but desire for knowledge was high. Our participants (particularly girls) felt that an educational component would be desirable, and presenting information about benefits of PA in a visual format was recommended. This is in line with our previous work suggesting less than 20% of parents knew the government PA guidelines for their children [31]. A review of 17 adolescent PA trials found that education alone did not result in behavior change. However, multicomponent studies incorporating education found strong effects on PA [10]. Multicomponent studies can be labor intensive (eg, requiring alteration of environmental infrastructure), so their potential for wide-scale implementation is questionable. The present *digital revolution* and the ubiquity and frequency of technology use by adolescents, highlighted in our study, has greatly increased potential reach.

Rewards were suggested as being important to encourage PA engagement (particularly in boys, but also for some girls), in line with previous studies [15,32]. The rewards suggested were always material (usually financial). Financial rewards are effective in motivating PA in adults [33], and a trial using incentives and gamification to promote PA in families had positive effects [34]. In line with theories such as the self-determination theory, fun and enjoyment are intrinsically motivating and key motivators for gaming [35], and most adolescents engage in gaming without a material reward. Fun was consistently reported as a reason for engaging in exergaming by our participants.

### Appeal of Home-Based Physical Activity

Our data suggest that a home-based PA digital intervention is appealing to our target population. Most previous interventions targeting adolescent PA have been at least partially delivered in schools, but a more recent cohort found 70% to 81% boys and girls said they would choose to be active at home or in a gym/leisure center, as opposed to in school or outdoors [32]. Particularly for the girls in our study, home-based PA had the potential to overcome cultural and social barriers. In addition, they were viewed as appealing because they harnessed behaviors (such as gaming) that were already being performed. Many suggested including additional intervention elements such as community-based meet ups or groups to enhance the social elements and foster competition. In the aforementioned quantitative survey, adolescents also reported they would choose to be active with their friends (over, eg, family) [32]. However, some girls in our study felt the ability to exercise privately in their own home was appealing, so an optional social element would be best.

### Potential for Virtual Reality Interventions

At the time of writing this paper, the cost of the high-end VR equipment required to create a fully immersive exergaming experience was prohibitive (around £2000 for the equipment and necessary computer), and this was recognized as a barrier by our participants. However, participants expect costs to fall. This is likely and ownership will increase [36]. We are aware that the novelty, reported as important in our study and others [15], will therefore also diminish. This strengthens the argument for developing a digital intervention within a theory-based framework, so the active ingredients can be replicated using other platforms in future. This also emphasizes the importance of making digital interventions intrinsically fun and enjoyable so that they are not reliant only on novelty. Indeed, in our study the participants who were most positive were those who had tried VR.

Our study had specific and narrow aims and around 60 min worth of discussion per participant. Interviews were conducted by researchers experienced in working with adolescents. Therefore, we believed our sample size held sufficient information power to address the research questions [37].

Our participants desired continual updates and additional challenge levels, and these are key elements of gaming that can map to specific behavior change techniques in a digital health intervention [38]. Embedding the digital platform at the center of a multicomponent intervention, and working with professional developers who understand how to keep a game engaging and challenging, is therefore crucial for success.

### Limitations

The results were obtained from a reasonable number of participants in comparison with other studies using thematic analysis [29], with a diverse range of ethnicities and more girls than boys. It is possible that those who were more interested in health agreed to participate. However, as less than 10% to 15% of the UK adolescent population meet PA recommendations [7], it is very unlikely that we only recruited active participants. The focus on technology as well as PA was also likely to attract a mix of interests. All participants were aged between 13 and 15 years, and results may have been different if opinions were from older adolescents. It is likely that our intervention will target younger adolescents given the value in intervening early (VR gaming is not recommended in those younger than 13 years). We will also continue to work with our public engagement groups and adolescent steering committee to gather information from adolescents from diverse backgrounds.

### Conclusions

The results of this study suggest that an intervention to promote PA in adolescents that has VR exergaming at the core is promising. However, it is likely that additional elements will be required to produce sustained behavior change including educational elements, tangible rewards, prompts to encourage breaks, and parental support.

## Abbreviations

PA: physical activity
PD: participatory design
VR: virtual reality

## References

[1] Warburton DE, Nicol CW, Bredin SS (2006). Health benefits of physical activity: the evidence. CMAJ.

[2] Arem H, Moore SC, Patel A, Hartge P, Berrington DG, Visvanathan K, Campbell PT, Freedman M, Weiderpass E, Adami HO, Linet MS, Lee I, Matthews CE (2015). Leisure time physical activity and mortality: a detailed pooled analysis of the dose-response relationship. JAMA Intern Med.

[3] Reiner M, Niermann C, Jekauc D, Woll A (2013). Long-term health benefits of physical activity--a systematic review of longitudinal studies. BMC Public Health.

[4] Telama R, Yang X, Viikari J, Välimäki I, Wanne O, Raitakari O (2005). Physical activity from childhood to adulthood: a 21-year tracking study. Am J Prev Med.

[5] Hallal PC, Victora CG, Azevedo MR, Wells JC (2006). Adolescent physical activity and health: a systematic review. Sports Med.

[6] Eime RM, Young JA, Harvey JT, Charity MJ, Payne WR (2013). A systematic review of the psychological and social benefits of participation in sport for children and adolescents: informing development of a conceptual model of health through sport. Int J Behav Nutr Phys Act.

[7] (2017). Health Survey for England.

[8] Kalman M, Inchley J, Sigmundova D, Iannotti RJ, Tynjälä JA, Hamrik Z, Haug E, Bucksch J (2015). Secular trends in moderate-to-vigorous physical activity in 32 countries from 2002 to 2010: a cross-national perspective. Eur J Public Health.

[9] Corder K, Sharp SJ, Atkin AJ, Griffin SJ, Jones AP, Ekelund U, van Sluijs EM (2014). Change in objectively measured physical activity during the transition to adolescence. Br J Sports Med.

[10] van Sluijs EM, McMinn AM, Griffin SJ (2007). Effectiveness of interventions to promote physical activity in children and adolescents: systematic review of controlled trials. Br Med J.

[11] Metcalf B, Henley W, Wilkin T (2012). Effectiveness of intervention on physical activity of children: systematic review and meta-analysis of controlled trials with objectively measured outcomes (EarlyBird 54). Br Med J.

[12] Rose T, Barker M, Maria JC, Morrison L, Lawrence W, Strömmer S, Vogel C, Woods-Townsend K, Farrell D, Inskip H, Baird J (2017). A systematic review of digital interventions for improving the diet and physical activity behaviors of adolescents. J Adolesc Health.

[13] Craig P, Dieppe P, Macintyre S, Michie S, Nazareth I, Petticrew M, Medical Research Council Guidance (2008). Developing and evaluating complex interventions: the new Medical Research Council guidance. Br Med J.

[14] Bartholomew LK, Parcel GS, Kok G (1998). Intervention mapping: a process for developing theory- and evidence-based health education programs. Health Educ Behav.

[15] Corder K, Schiff A, Kesten JM, van Slujis EM (2015). Development of a universal approach to increase physical activity among adolescents: the GoActive intervention. BMJ Open.

[16] DeSmet A, Thompson D, Baranowski T, Palmeira A, Verloigne M, de Bourdeaudhuij I (2016). Is participatory design associated with the effectiveness of serious digital games for healthy lifestyle promotion? A meta-analysis. J Med Internet Res.

[17] Granic I, Lobel A, Engels RC (2014). The benefits of playing video games. Am Psychol.

[18] (2019). Nintendo.

[19] Pocketgamer.

[20] Ma BD, Ng SL, Schwanen T, Zacharias J, Zhou M, Kawachi I, Sun G (2018). Pokémon GO and physical activity in Asia: multilevel study. J Med Internet Res.

[21] Gao Z, Chen S, Pasco D, Pope Z (2015). A meta-analysis of active video games on health outcomes among children and adolescents. Obes Rev.

[22] Staiano AE, Abraham AA, Calvert SL (2013). Adolescent exergame play for weight loss and psychosocial improvement: a controlled physical activity intervention. Obesity (Silver Spring).

[23] Staiano AE, Marker AM, Beyl RA, Hsia DS, Katzmarzyk PT, Newton RL (2017). A randomized controlled trial of dance exergaming for exercise training in overweight and obese adolescent girls. Pediatr Obes.

[24] Csikszentmihalyi M (1975). Beyond Boredom and Anxiety: Experiencing Flow in Work and Play.

[25] Slater M, Wilbur S (1997). A framework for immersive virtual environments (FIVE): speculations on the role of presence in virtual environments. Presence (Camb).

[26] Zeng N, Pope Z, Gao Z (2017). Acute effect of virtual reality exercise bike games on college students' physiological and psychological outcomes. Cyberpsychol Behav Soc Netw.

[27] Murray EG, Neumann DL, Moffitt RL, Thomas PR (2016). The effects of the presence of others during a rowing exercise in a virtual reality environment. Psychol Sport Exer.

[28] Finkelstein S, Suma EA (2011). Astrojumper: Motivating exercise with an immersive virtual reality exergame. Presence (Camb).

[29] Fugard AJ, Potts HW (2015). Supporting thinking on sample sizes for thematic analyses: a quantitative tool. Int J Soc Res Methodol.

[30] Gale NK, Heath G, Cameron E, Rashid S, Redwood S (2013). Using the framework method for the analysis of qualitative data in multi-disciplinary health research. BMC Med Res Methodol.

[31] Sawyer A, Smith L, Schrempft S, van Jaarsveld CH, Wardle J, Fisher A (2014). Primary caregiver knowledge of paediatric physical activity recommendations in the United Kingdom and its association with caregiver behaviour: an observational study. BMC Public Health.

[32] Corder K, Atkin AJ, Ekelund U, van Sluijs EM (2013). What do adolescents want in order to become more active?. BMC Public Health.

[33] Barte JC, Wendel-Vos GC (2017). A systematic review of financial incentives for physical activity: The effects on physical activity and related outcomes. Behav Med.

[34] Zimmerman FJ (2009). Using behavioral economics to promote physical activity. Prev Med.

[35] Ryan RM, Rigby CS, Przybylski A (2006). The motivational pull of video games: a self-determination theory approach. Motiv Emot.

[36] Avila L, Bailey M (2014). Virtual reality for the masses. IEEE Comput Grap Appl.

[37] Malterud K, Siersma VD, Guassora AD (2015). Sample size in qualitative interview studies: guided by information power. Qual Health Res.

[38] Cugelman B (2013). Gamification: what it is and why it matters to digital health behavior change developers. JMIR Serious Games.

