# Title Correction: Younger Adolescents’ Perceptions of Physical Activity, Exergaming, and Virtual Reality: Qualitative Intervention Development Study

**DOI:** 10.2196/15657

**Published:** 2019-09-13

**Authors:** Nuša Faric, Eleanor Yorke, Laura Varnes, Katie Newby, Henry WW Potts, Lee Smith, Adrian Hon, Andrew Steptoe, Abigail Fisher

**Affiliations:** 1 Department of Behavioural Science & Health University College London London United Kingdom; 2 Faculty Research Centre for Advances in Behavioural Science Coventry University Coventry United Kingdom; 3 Institute of Health Informatics University College London London United Kingdom; 4 Cambridge Centre for Sport and Exercise Sciences Anglia Ruskin University Cambridge United Kingdom; 5 Six to Start London United Kingdom

The title of this paper (JMIR Serious Games 2019;7(2):e11960) has been changed from “Younger Adolescents’ Perceptions of Physical Activity, Exergaming, and Virtual Reality: Qualitative Intervention Study” to “Younger Adolescents’ Perceptions of Physical Activity, Exergaming, and Virtual Reality: Qualitative Intervention Development Study”.

The corrections will appear in the online version of the paper on the JMIR website on September 13, 2019, together with the publication of this correction notice. Because this was made after submission to PubMed, PubMed Central, and other full-text repositories, the corrected article also has been resubmitted to those repositories.

